# Isolated retinal astrocytic hamartoma with 7-year follow-up: A case report

**DOI:** 10.1097/MD.0000000000034522

**Published:** 2023-09-01

**Authors:** Bogumiła Wójcik-Niklewska, Sebastian Sirek, Agnieszka Tronina, Erita Filipek

**Affiliations:** a Department of Pediatric Ophtalmology, Faculty of Medical Sciences in Katowice, Medical University of Silesia in Katowice, Katowice, Poland; b Kornel Gibiński University Clinical Centre, Katowice, Poland; c Department of Ophthalmology, Faculty of Medical Sciences in Katowice, Medical University of Silesia in Katowice, Katowice, Poland.

**Keywords:** astrocytic cells, benign tumor, retinal astrocytic hamartoma

## Abstract

**Rationale:**

Retinal astrocytic hamartoma (RAH) is a rare benign tumor originating from astrocytic cells located in the neural cell layer of the retina. It is commonly seen in patients with phakomatoses such as tuberous sclerosis complex or neurofibromatosis, rarely as an isolated retinal mass. This lesion is usually asymptomatic; however, these located in the area of the optic nerve, macula, or exhibiting the features of exudation, neovascularization may present visual disturbances and decreased visual acuity.

**Patient concerns:**

We present a rare case of a 15-year-old boy, with no significant past medical history, whose cause of visual disturbances turned out to be isolated RAH.

**Diagnoses:**

Based on the results of color images of the fundus, fluorescein angiography as well as the analysis of magnetic resonance imaging, the patient was diagnosed with RAH.

**Interventions:**

Additionally an B-scan ultrasonography, static and kinetic perimetry were performed.

**Outcomes:**

Fundoscopic examination showed a unilateral yellowish, well-circumscribed, mulberry-like lesion with a wide base, located in inferosnasal quadrant, in the vinicity of the optic nerve. The patient underwent neurological, pediatric, and genetic evaluations that excluded other pathological findings or underlying systemic disease.

**Lessons:**

The prognosis for RAH is generally good, however, the lesion requires regular ophthalmologic follow-up to rule out the progression of the tumor mass. The patient 7-year follow-up history is without evidence of tumor growth, local or general deterioration of the condition.

## 1. Introduction

Retinal astrocytic hamartoma (RAH) is a rare benign neoplasm composed of glial cells located in the nerve fiber layer of the retina. This tumor is part of the retinal astrocytic tumor family that includes other, not always nonprogressive lesions from which it must be distinguished. RAH is usually associated with a systemic disease, either tuberous sclerosis complex (TSC) or neurofibromatosis (NF). Fundus examination reveals bilateral RAH in approximately 50% of patients with TSC. It rarely occurs as an isolated lesion, is usually an incidental finding during ophthalmic examination diagnosed later in life.^[1–3]^

The majority of RAHs are endophytic tumors growing toward the vitreous, but some can be endophytic subretinal lesions developing in retinal nerve fibers. Histopathologically, it is an eosinophilic lesion, made up of well-circumscribed, elongated fibrous astrocytes, with eosinophilic cytoplasm and round or oval nucleus. The tumor can be located peripherally or in the peripapillary area, and can be a yellowish, slightly elevated lesion; in some patients the nodule can be semitranslucent with central calcification. The majority of astrocytic hamartomas are slow-growing tumors that tend to calcify over time. They need to be differentiated from retinoblastoma, acquired retinal astrocytoma, reactive retinal astrocytic tumor or amelanotic choroid melanoma.^[4–6]^

## 2. Case report

**Figure 1.**
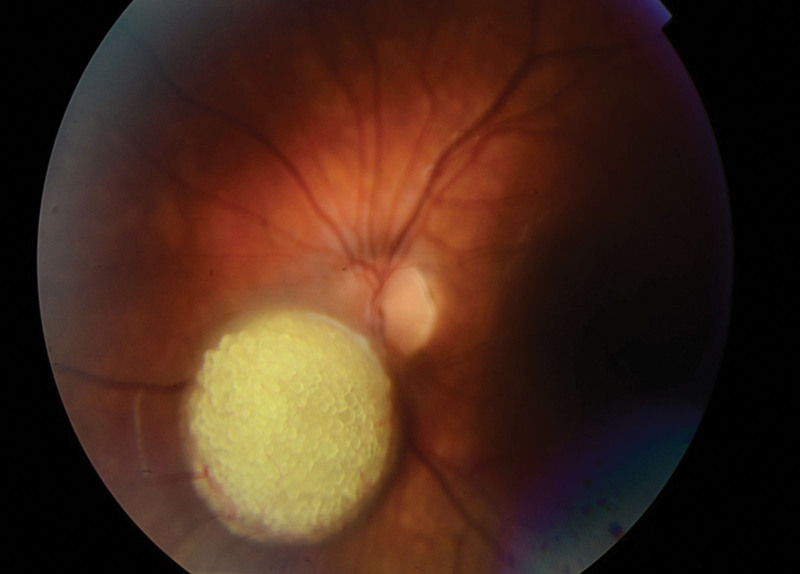
Fundoscopic appearance of an left eye - (initial examination).

**Figure 2.**
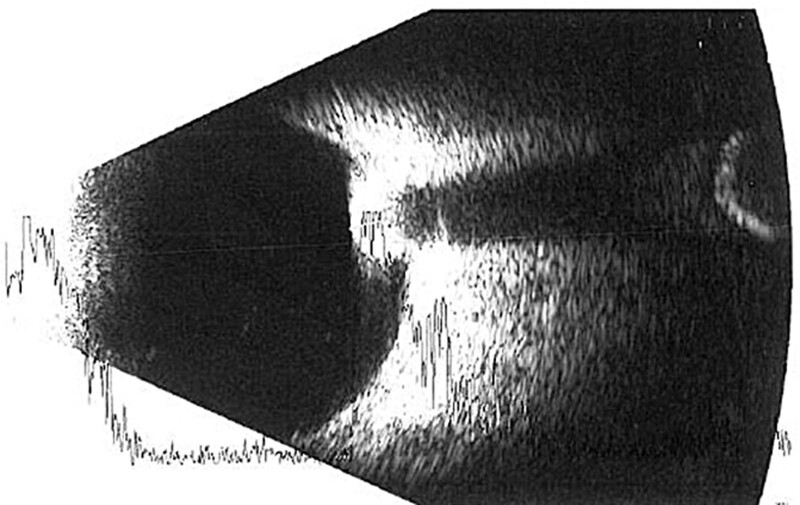
Left eye ultrasonography.

**Figure 3.**
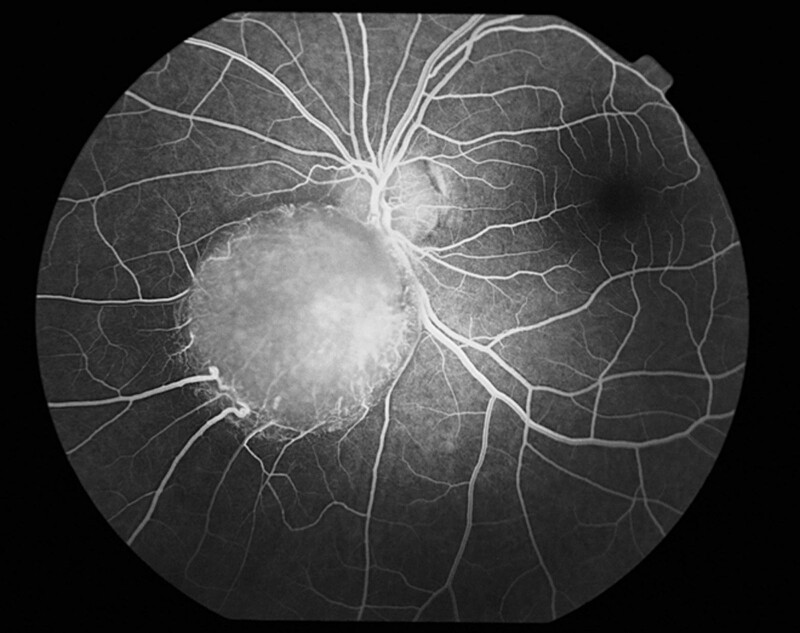
Fluorescein angiography of the left eye fundus.

**Figure 4.**
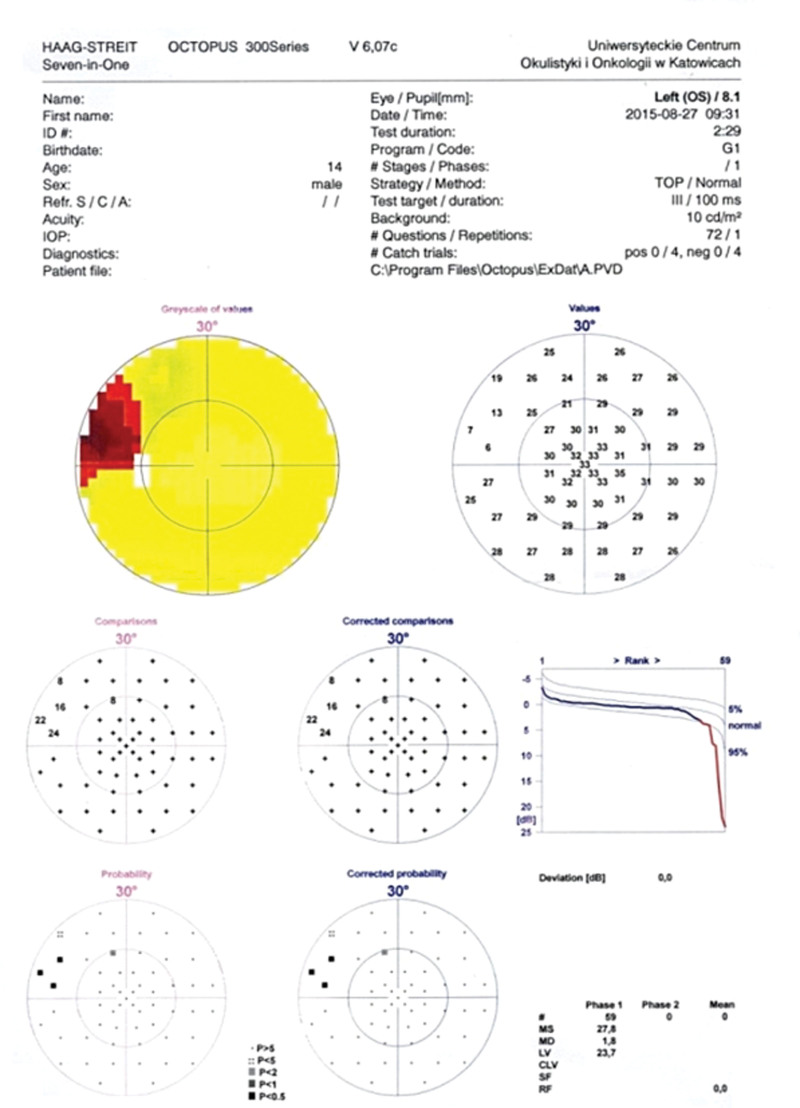
Left eye static visual field.

**Figure 5.**
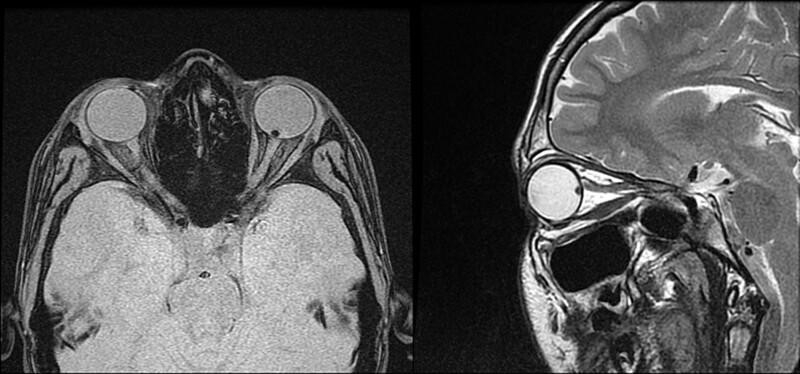
Magnetic resonance imaging of the head and orbits.

**Figure 6.**
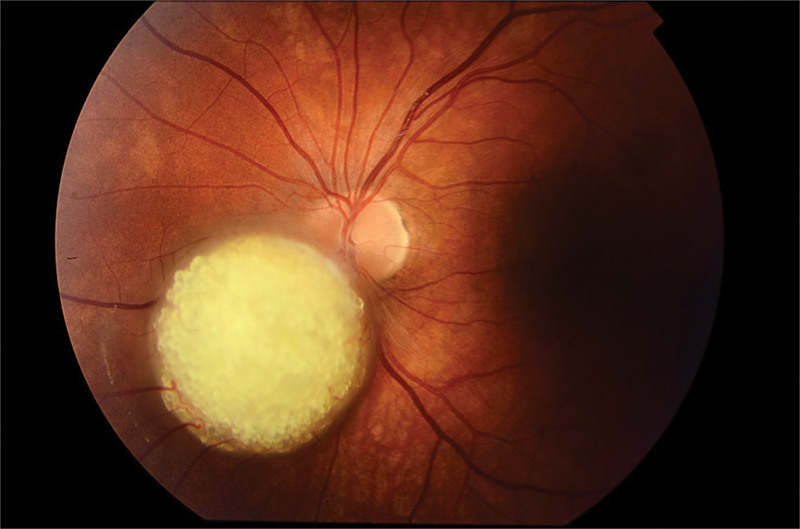
Fundoscopic appearance of the left eye - 7-years follow-up.

**Figure 7.**
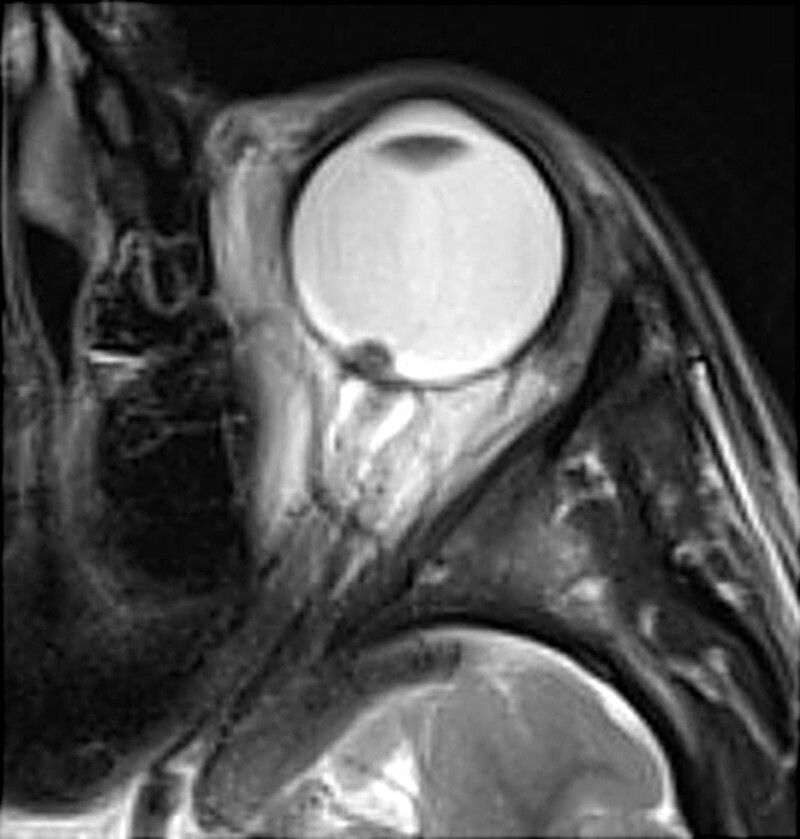
Magnetic resonance imaging of the head and orbits - 7-years follow-up.

A 15-year-old boy was referred to the Department of Pediatric Ophthalmology, the K. Gibiśski University Hospital Center, Medical University of Silesia, Katowice, Poland due to decline in distance visual acuity. His medical and family history did not reveal any eye disease or concomitant diseases. The eyeballs were normally positioned within orbit, with full mobility range and preserved binocular vision. Best-corrected visual acuity of the right eye was 1.0 with spherical correction of −0.75 and cylinder correction of −0.25 (axis 100º), and of the left eye 1.0 with spherical correction of −1.25. Near vision was within normal range. Intraocular pressure measured with the Goldmann tonometer was 15 mm Hg in both eyes. No abnormalities were detected in anterior segments of right or left eye. Fundocopic examination of the left eye revealed a yellowish, mulberry-like retinal mass with a wide base located in lower nasal quadrant, adjacent to the optic nerve disc (Fig. 1), while right eye fundus was without any pathological findings. Further diagnostic testing was conducted to determine the nature of the lesion. Left eyeball ultrasound showed a hyperechoic lesion (2.7 mm × 5.7 mm) adjacent to the optic nerve disc, protruding into vitrous body (Fig. 2). The arterial phase of fluorescein angiography showed a surface capillary network around the tumor, with evidence of fluorescein retention (Fig. 3). Static perimetry showed an absolute central scotoma in the upper temporal quadrant of left eye visual field, corresponding to the anatomical location of the tumor, while kinetic perimetry did not reveal any vision field alterations (Fig. 4). Evaluation the sectorial thickness of single retinal layers and optic nerve was performed with spectral domain optic coherence tomography. It reveals the transition from a normal retina to a hyperreflective tumor mass that was confined to the outer retinal layers, with insignificant surrounding edema and posterior shadowing. There was no evidence of calcification or retinal traction. Magnetic resonance imaging (MRI) of the head and left orbit showed a 3.8 × 2.5 mm area of low-signal intensity on all sequences of the lower pole of the optic nerve disc. The eyeballs were of normal size, shape and contour, normally located, with full mobility range. There was no enlargement or pathological signals from the extraocular muscles. Optic nerves, optic chiasm and optic tracts were normal. The volume of orbital adipose tissue was also normal and so were lacrimal glands. No enhancement was seen after contrast administration (Fig. 5). Consultations by a pediatricians and neurologist ruled out systemic diseases, TSC or NF; head MRI did not reveal any abnormalities of the central nervous system. The patient undergoes regular checkups by neurologists and neurosurgeons. A 7-year ophthalmic follow-up does not show any progress of the fundus lesion in the left eye; visual acuity does not worsen either. On the last examination, best-corrected visual acuity of left and right eye was 1.0; near vision was not deteriorated. Intraocular pressure was 16 mm Hg in both eyes. No anterior segments or right fundus abnormalities were found. Left fundus images showed the same retinal tumor with some features of calcification adjacent to the optic nerve disc, its size being comparable to previous examinations (Fig. 6). Follow-up static and kinetic perimetry confirmed an absolute scotoma corresponding to the anatomical tumor location. Head and left orbit MRI did not reveal any abnormalities except for a low-signal area in the lower pole of the optic nerve disc and calcifications within mass with no contrast enhancement (Fig. 7).

## 3. Discussion

RAH is a benign tumor that infrequently reduces visual acuity and does not require treatment. It arises from astrocytes being an integral part neural retinal. Astrocytes are glial cells, providing support for retinal ganglionic cells, maintaining functional neuronal circuits and being part of the mechanism leading to reactive gliosis. These cells are located in the inner layers of the retina, where they migrate independently of blood vessels. Thus, they are involved in the retinal response to hypoxia and mediate the production of vascular endothelial growth factor.^[7]^ Tumor that originate from astrocytes in the retina is called retinal astrocytic tumor. According to Rowley at al. they we differentiate its 3 subtypes.^[8,9]^ Although they may share many similarities, particularly in fundoscopic appearance, each has its own unique morphologic and histologic characteristics, possibility of pathological progression and some are closely related to underlying primary disease. Reactive retinal astrocytic tumor is typically observed in the third decade of life and is neither genetic nor hereditary. It is thought to occur in response to retinal inflammation, degeneration or injury. Lesion is usually unilateral, single, peripherally located and has a venous component that gives it a yellow-pink tint. It is a progressive tumor with a high incidence of exudates that lead to retinal detachment.^[10,11]^ Acquired retinal astrocytoma is also a solitary, benign lesion but may show locally aggressive features. It is not associated with TSC and is distinguished by a yellowish color related to numerous lipid exudates. This tumor can lead to a variety of intraocular complications including vitreous hemorrhages, retinal detachment and secondary glaucoma. Its precise and accurate diagnosis is crucial since this type typically requires prompt treatment, recently verteporfin photodynamic therapy has been recommended.^[12]^ RAH is usually a whitish-yellow lesion and is extremely often seen as part of TSC. Other clinical entities in which RAH has been observed include NF, Stargardt’s disease, Retinitis pigmentosa, and Usher’s syndrome. It constitutes one of the minor criteria for the diagnosis of TSC and it is also the most frequent retinal lesion observed in patients with this disease. When associated with TSC, the tumor is usually bilateral and multifocal.^[13]^ Typical RAH is described as single, flat, and semiopaque lesion. It is usually diagnosed in childhood and shows features of calcification. Progression of these lesions is not reported–it usually remains stable and does not require inclusion of treatment. Visual field restriction and a decline in visual acuity might be found depending on lesion location and potential pressure on the optic nerve. Clinical signs of retinal astrocytoma associated with tuberous sclerosis progress slowly. Zimmer-Galler et al^[14]^ examined 16 patients with a total of 37 RAHs. During follow-up that ranged from 6 to 34 years only 3 lesions showed progressive or new calcifications. Shields et al^[15]^ observed that peripheral astrocytomas did not exhibit growth during follow-up while those located near the optic disc did. Fast-growing lesions may cause retinal detachment, surrounding tissue necrosis, inflammatory response, macular edema, retinal vein occlusion, vitreous body hemorrhages or neovascular glaucoma and requires specific treatment^[16]^ To date, no cases of RAH evolving into a malignant form or generating metastases have been described.^[17]^

We distinguish 3 subtypes of RAH subtypes based on morphological features. Type 1 is a small, flat, isolated, grayish lesion that is semitranslucent, with no evidence of calcification. Conversely, there is type II which is defined by its yellowish color and mulberries-like shape. It is nodular, modular and has many calcifications. It is nodular, contains multiple calcifications and is raised in relation to the retinal surface. Type 3 exhibits the characteristics of both of the previous 2.^[8,18]^ Based on this classification, our case falls under type 2.

Differential diagnosis is always needed to exclude retinal malignancies. The most common lesions to be confused with are retinoblastoma, choroid uveal melanoma, and choroidal metastasis. Significant features which distinguish RAH from retinoblastoma are calcifications of a yellow tone and the absence of veins protruding into the lesion. Uveal melanoma has a resemblance of cavitation but rarely presents with calcifications. Choroidal metastasis is usually detected in older patients, usually with breast or lung cancer. They are distinguished by yellow masses located underneath the retina with large subretinal exudates that cause retinal detachment and consequently a decrease in visual acuity in 50% of cases.^[19]^

High prevalence of astrocytoma among patients with tuberous sclerosis indicates a need for evaluation by internists to exclude characteristic skin lesions, kidney tumors, heart tumors, subependymal nodules and subpleural cysts.^[3]^ Retinal lesions develop within 2 to 6 years of TSC diagnosis in approximately 80% of patients with tuberous sclerosis, but can also be the first clinical symptom of the disease.^[7]^ Patients should undergo regular ophthalmic and neurological follow-ups. Each patient with RAH should undergo a thorough diagnostic process, including differential diagnosis. They also need regular follow-up examinations (every 3 to 6 months) and specialist consultations to rule out systemic diseases.^[20]^

## 4. Conclusion

This case illustrates the importance of recognizing different types of retinal astrocytic lesions that may be associated with various clinical conditions and have different potential for malignancy. The lack of calcifications, the whitish-yellow tint of the lesion, and additional tests that ruled out TSC indicated a different type of tumor than the 1 we initially suspected. However, careful examination, frequent and regular follow-ups allowed us to observe changes that gave the tumor a clear morphologic picture, which is type 2 astrocytic retinal hamartoma. At the same time, the lesion did not change in nature, did not enlarge, did not cause complications, and most importantly did not decrease visual acuity, which was observed throughout the 7-year follow-up period.

## Acknowledgments

The authors are grateful to the patient and his family for their suport of this article.

## Author contributions

**Conceptualization:** Sebastian Sirek, Agnieszka Tronina, Erita Filipek.

**Data curation:** Bogumiła Wójcik-Niklewska, Sebastian Sirek, Agnieszka Tronina.

**Formal analysis:** Bogumiła Wójcik-Niklewska, Sebastian Sirek, Agnieszka Tronina.

**Investigation:** Bogumiła Wójcik-Niklewska, Sebastian Sirek, Agnieszka Tronina.

**Methodology:** Bogumiła Wójcik-Niklewska, Sebastian Sirek, Agnieszka Tronina, Erita Filipek.

**Project administration:** Bogumiła Wójcik-Niklewska, Erita Filipek.

**Resources:** Bogumiła Wójcik-Niklewska.

**Software:** Bogumiła Wójcik-Niklewska.

**Supervision:** Bogumiła Wójcik-Niklewska, Erita Filipek.

**Validation:** Bogumiła Wójcik-Niklewska.

**Visualization:** Bogumiła Wójcik-Niklewska, Sebastian Sirek.

**Writing – original draft:** Sebastian Sirek.

**Writing – review & editing:** Bogumiła Wójcik-Niklewska, Sebastian Sirek.
